# Pregnancy outcomes in women with severe acute respiratory syndrome coronavirus 2 reinfections compared to those with a single infection: a retrospective cohort study

**DOI:** 10.1186/s12884-024-06657-y

**Published:** 2024-07-03

**Authors:** Yan Ma, Qingxia Zhang, Zhenli Shan, Yanting Chen, Yan Chen, Xiaoyu Pan, Yiying Huang

**Affiliations:** 1 Shanghai Key Laboratory of Maternal Fetal Medicine, Shanghai Institute of Maternal-Fetal Medicine and Gynecologic Oncology, Shanghai First Maternity and Infant Hospital, School of Medicine, Tongji University, 536 Changle Road, Jingan District, Shanghai, 200040 China; 2 Department of Obstetrics and Gynecology, China-Japan Friendship Hospital, 2 Yinghuayuan East Streat, Chaoyang District, 100029 Beijing, China; 3 Department of Obstetrics and Gynecology, North China University of Science And Technology Affiliated Hospital, 063099 Tangshan, Hebei Province China

**Keywords:** Coronavirus disease 2019, Reinfection, Pregnancy outcome, Gestation trimester

## Abstract

**Background:**

To assess pregnancy outcomes in women with severe acute respiratory syndrome coronavirus 2 (SARS-CoV-2) reinfection.

**Methods:**

This was a retrospective cohort study that included pregnant women who contracted coronavirus disease 2019 (COVID-19) once or twice during pregnancy and who gave birth between 1 October 2022 and 15 August 2023 in Shanghai First Maternity and Infant Hospital (Shanghai, China). We collected their clinical data and compared the frequency of adverse pregnancy outcomes between the reinfection group and the primary infection group, such as preterm birth, fetal growth restriction (FGR), hypertensive disorders of pregnancy (HDP), common pregnancy-related conditions, birth weight, and neonatal unit admission.

**Results:**

We observed a 7.7% reinfection rate among the 1,405 women who contracted COVID-19 during pregnancy. There were no significant differences in the frequency of preterm birth, FGR, HDP, other common pregnancy-related conditions, birth weight, or rate of neonatal unit admission between the reinfection and single infection groups. All our participants were unvaccinated, and all had mild symptoms.

**Conclusion:**

Our study showed no significant association between SARS-CoV-2 reinfection and adverse pregnancy outcomes.

## Background

Since the onset of the coronavirus disease 2019 (COVID-19) pandemic, many studies have evaluated the association between COVID-19 in pregnancy and maternal and fetal outcomes [1–3]. Compared to the general population, pregnant women are more susceptible to COVID-19, more likely to develop severe illness and sequelae, and at a higher risk of dying or requiring admission to an intensive care unit [4]. An international registry study demonstrated an increase in adverse maternal and neonatal outcomes in women who contracted COVID-19 during pregnancy [5]. Another national study demonstrated that pregnant women infected with severe acute respiratory syndrome coronavirus 2 (SARS-CoV-2) experienced higher rates of fetal death, preterm birth, preeclampsia, emergency caesarean delivery, and prolonged maternal and neonatal hospital stays after birth than those not infected with SARS-CoV-2 [6]. Several studies have associated the gestational age at time of SARS-CoV-2, SARS-CoV-2 variant, vaccination status, and herd immunity, with pregnancy outcomes [7–9].

On 25 August 2020, the first case of reinfection by a phylogenetically distinct variant of SARS-CoV-2 was reported in the medical literature [10]. Reinfection was defined as the appearance of new symptoms suggestive of COVID-19 upon medical evaluation and confirmed by a positive reverse transcription-polymerase chain reaction (RT-PCR) test for SARS-CoV-2, occurring > 90 days after a previous RT-PCR-confirmed infection [11]. However, as persistent shedding of viral RNA from the primary infection can also cause repeated positive RT-PCR tests, confirmed cases of SARS-CoV-2 reinfection remain relatively rare. It is recommended that genetic testing is performed, and its results should be evaluated alongside cycle threshold (Ct) values and clinical status to confirm reinfection [12]. A study in Qatar compared two groups of unvaccinated people: almost 6000 who had contracted the disease once and 1300 who had been reinfected. The results of the study showed that the odds of developing a severe, critical, or fatal disease after reinfection were almost 90% lower in those reinfected than in those with primary infection [13]. Another study reported that reinfection further increases risks of death, hospitalization, and sequelae in multiple organ systems in the acute and post-acute phases [14]. However, the clinical consequences of reinfection during pregnancy remain unknown. This study, therefore, aimed to investigate the effects of maternal SARS-CoV-2 reinfection during pregnancy on maternal and fetal outcomes.

## Methods

This single-center, retrospective study was conducted at Shanghai First Maternity and Infant Hospital (Shanghai, China) between 1 October 2022 and 15 August 2023. A total of 108 pregnant women who gave birth at our hospital and had SARS-CoV-2 infections twice were included in the study. COVID-19 cases were confirmed with RT-PCR or antigen testing following product guidance specific for each test and platform. SARS-CoV-2 RT-PCR result was considered positive with an amplification Ct ≤ 38. Reinfection was determined in accordance with the “90-day” guidance from the Centers for Disease Control and Prevention (CDC). Patients with only one positive SARS-CoV-2 test result during pregnancy and no previous infection who gave birth within the same time period at the same hospital were included in the comparison group. These patients were classified into three groups according to which trimester the infection occurred in: <14 weeks of gestation (first trimester), 14–28 weeks of gestation (second trimester), and > 28 weeks of gestation (third trimester). The SARS-CoV-2 variant was not determined in our hospital due to a lack of equipment and technology.

The timing, symptoms, vaccination status, and test results of COVID-19 were reported by participants during the postpartum examination or via phone calls. According to the criteria described by Wu et al., [15] pregnant women in our study all had mild COVID-19 symptoms. The medical records of the patients were reviewed, and data on maternal demographic and obstetric characteristics and maternal and fetal outcomes were collected and analyzed. Deliveries that occurred at < 20 weeks of gestation were excluded. None of the patients included in our study were vaccinated against COVID-19 at the time of infection. All procedures were approved by the Ethics Committee of Shanghai First Maternity and Infant Hospital (No. KS23312). The requirement for informed consent for data collection was waived because of the retrospective nature of the study.

Stillbirth was defined as pregnancy loss at ≥ 20 weeks of gestation. Fetal growth percentiles were calculated with gestational age and weight based on World Health Organisation fetal growth charts [16]. Fetal growth restriction (FGR) was diagnosed in babies in the bottom 10th percentile at birth. Preterm birth was defined as birth before gestational week 37, as determined from the medical records. Hypertensive disorders of pregnancy (HDP) were diagnosed in accordance with the guidelines of the International Society for the Study of Hypertension in Pregnancy [17].

The primary outcomes assessed included preterm birth, FGR, HDP, common pregnancy-related conditions, birth weight, and neonatal unit admission. Secondary outcomes were the duration and symptoms of COVID-19 during pregnancy.

It was reported previously that the odds ratio (OR) of preterm birth in women with SARS-COV-2 infection is approximately 2.0. The preterm rate in pregnant women with SARS-COV-2 infection is approximately 17% [18]. With a level of statistical significance of 0.05 and a power of 0.8, it was estimated that a sample size of 102 women with reinfection was needed in the case group. We enrolled 108 pregnant women with SARS-CoV-2 reinfection, and the control group consisted of 1297 women with SARS-CoV-2 primary infection (single infection) based on a 1:12 case-to-control ratio.

### Statistical analysis

Data analysis was performed using SPSS software version 23.0 (IBM Corp., Armonk, NY, USA). Data are presented as mean ± standard deviation, or number and frequency. Continuous variables were compared using analysis of variance. Fisher’s exact test or chi-squared test was used to compare categorical variables. Differences were considered significant when the *p*-value was < 0.05.

## Results

**Fig. 1:**
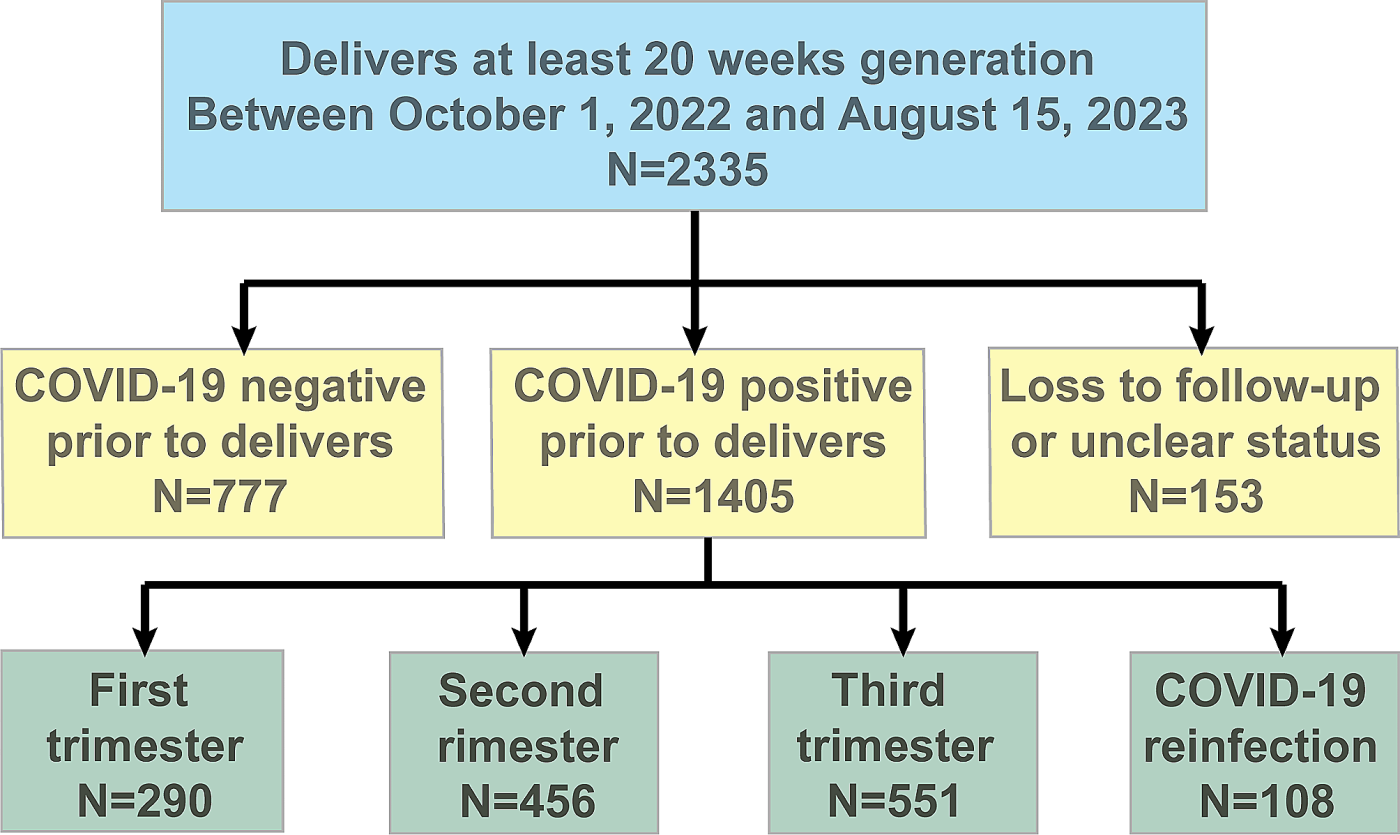
The flow chart of study

Between 1 October 2022 and 15 August 2023, 2,335 pregnant women gave birth at Shanghai Maternal and Infant Hospital, with 1405 of them identified as having had a SARS-CoV-2 infection during pregnancy. We identified 108 pregnant women with positive RT-PCR or antigen tests ≥ 90 days after an initial positive test accompanied by compatible symptoms and known exposure to the virus. The remaining 1297 patients had a single positive test: first trimester, *n* = 290; second trimester, *n* = 456; third trimester, *n* = 551 (Fig. 1: The flow chart of study). Pregnant women with a positive SARS-CoV-2 test within 90 days of an initial positive test were deemed as having an uncertain status and excluded from the study.

The reinfection rate during the study period was 7.7% (108/1405), with an average 150-day interval (90–224 days) between positive tests. Reinfections occurred 3–7 months after the resolution of the first infection. The characteristics of the study participants are reported in Table 1. Among the women who were reinfected, 23 were diagnosed with COVID-19 in the first and second trimesters, 61 were diagnosed in the first and third trimesters, and 24 were diagnosed in the second and third trimesters.

**Table 1:** Demographics and comorbidities of women included in study

	Trimester became COVID-19 positive in primary infection group *n* = 1297			COVID-19 reinfection	*P*
	first trimester	second trimester	third trimester		
	*n* = 290	*n* = 456	*n* = 551	*n* = 108	
Age(y)	31.8 ± 4.01	31.7 ± 3.91	31.9 ± 3.80	31.8 ± 3.74	0.973
BMI(kg/m2)	21.9 ± 3.28	21.6 ± 3.03	21.3 ± 3.03	22.0 ± 3.05	0.062
Primiparity	222(76.6%)	340(74.6%)	407(73.9%)	80(74.1%)	0.851
ART conception	31(10.7%)	34(7.5%)	21(3.8%)	7(6.5%)	0.967
Previous preterm birth	3(1.0%)	4(0.9%)	2(0.4%)	0	
Multiple pregnancy	3(1.0%)	4(0.9%)	2(0.4%)	3(2.8%)	0.057
Previous hypertensive disorder of pregnancy	0	2(0.4%)	0	0	
Previous cesarean delivery	24(8.3%)	42(9.2%)	43(7.8%)	14(13.0%)	0.102
Pregestational diabetes mellitus	1(0.3%)	2(0.4%)	2(0.4%)	0	
Chronic hypertension	3((1.0%)	3(0.7%)	6(1.1%)	0	
Thyroid disease	19(6.6%)	29(6.4%)	36(6.5%)	7(6.5%)	0.984

ART: assisted reproductive technology

There were no statistically significant differences in age, body mass index (BMI), or primiparity frequency between the reinfection and single infection groups (Table 1), but the median BMI (22) in the reinfection group was slightly higher than those in the other groups. No cases of preexisting diabetes or hypertension were observed in the reinfection group. Thyroid disease was the most frequently observed comorbidity across the groups. The reinfection and single infection groups had comparable rates of assisted reproductive technology (ART) conception, multiple pregnancies, previous preterm birth, and previous caesarean delivery.

Reported symptoms of COVID-19 included fever, cough, stuffy/running nose, sore throat, fatigue, headache, nausea and vomiting, myalgia, liver dysfunction, heart palpitations, and loss of taste or smell (Table 2). Pregnant women reinfected with SARS-CoV-2 presented with milder symptoms compared to women experiencing an initial infection. There were significant differences in the rates of fever between the two groups, with fever being more common in the first infection group (*P* < 0.001). Individuals who experienced reinfection recovered from the second infection more quickly (4.14 ± 0.17 days) than those who experienced a single infection (7.26 ± 0.07 days; *p* < 0.001). A total of 27 women were diagnosed with reinfection based on the antigen test, while 81 women were diagnosed with reinfection with a positive RT-PCR. In the control group,146 women were diagnosed with the antigen test and 1151 women with RT-PCR.

**Table 2:** Symptom and distribution of SARS-CoV-2 tests in COVID-19 infection women

Symptom		Reinfection(*n* = 108)		Primary infection(*n* = 1297)		OR	CI95%	*P*
		N	%	N	%			
Fever		53	49.1	1118	86.2	6.48	4.31–9.76	0
Cough		43	39.8	526	40.6	1.03	0.69–1.54	0.88
Stuffy running nose		25	23.1	247	19	0.78	0.49–1.25	0.3
Myalgia		20	18.5	325	25.1	1.47	0.89–2.43	0.13
Sore throat		27	25	229	17.7	0.64	0.41–1.42	0.06
Nause/vomiting		7	6.5	63	4.9	0.74	0.33–1.65	0.46
Loss of taste or smell		9	8.3	83	6.4	0.75	0.37–1.54	0.44
Headache		16	14.8	214	16.5	1.14	0.66–1.97	0.65
Fatigue		16	14.8	188	14.5	0.98	0.56–1.69	0.93
Heart palpitation		1	0.9	12	0.9	1	0.13–7.76	1
liver dysfunction		0		23				
		mean	SD	mean	SD	df	F value	P
Infection duration		4.14	0.17	7.26	0.07	1	177.99	0
Distribution of SARS-CoV-2 tests	Antigen test	27/216	12.5	146/1297	11.3	1.13	0.73–1.75	0.6
	RT-PCR test	189/216	87.5	1151/1297	88.7			

df: degree of freedom

After adjusting for age, BMI, ART conception, multiple pregnancies, nullipara, and chronic hypertension in the reinfection and control groups, our study revealed that SARS-CoV-2 reinfection was not related to preterm delivery, HDP, or FGR (Table 3). In the refection and control groups, the preterm birth rates were 3.7% and 5.2% (*P* = 0.66, adjusted RR [aRR] = 1.29, 95% confidence interval [CI] = 0.40–4.13), the incidence rates of HDP were 6.5% and 7.4% (*P* = 0.58, aRR 1.24, 95% CI = 0.58–2.67), and the FGR rates were 1.9% and 2.0% (*P* = 0.88, aRR 1.13, 95% CI = 0.25-5.00), respectively. There were no significant differences between the reinfection and single infection groups in the frequencies of gestational diabetes (GDM), intrahepatic cholestasis of pregnancy (ICP), liquor volume abnormality, placenta previa, postpartum hemorrhage, deep vein thrombosis (DVT), or pulmonary embolism (PE). A comparison of perinatal outcomes between the groups yielded no significant differences in birth weight, stillbirth rate, or neonatal unit admission rate.

**Table 3:** Rate and risks of complications of COVIID-19 reinfection and primary infection group

	COVID-19 diagnosis				Univariable analysis					Multivariable analysis		
	Primary infection		Reinfection									
	*N* = 1297	%	*N* = 108	%	OR	CI95%	P	aOR	CI95%			P
Preterm birth < 37wk	68	5.2	4	3.7	1.439	0.515–4.022	0.486	1.291	0.404		4.126	0.666
Fetal growth restriction	26	2	2	1.9	1.084	0.254–4.63	0.913	1.125	0.253		5.004	0.877
Hypertensive disorders of pregnancy	84	6.5	8	7.4	0.866	0.408–1.839	0.707	1.242	0.579		2.665	0.578
Gestational diabetes	135	10.4	11	10.2	1.025	0.536–1.961	0.94	1.006	0.532		1.900	0.986
ICP	23	1.8	1	0.9	1.932	0.258–14.443	1	0.572	0.101		3.236	0.528
Liquor volume abnormality	37	2.9	5	4.6	0.605	0.233–1.574	0.369	1.727	0.686		4.347	0.246
Placenta praevia	16	1.2	1	0.9	1.336	0.176–10.175	1	1.063	0.203		5.576	0.943
Postpartum hemorrhage	13	1	1	0.9	1.083	0.14–8.361	1	0.899	0.147		5.509	0.908
DVT or PE	8	0.6	2	1.9	0.329	0.069–1.569	0.176	4.009	0.879		18.292	0.073
Live birth	1295		109		/	/	/					
Still birth	6	0.5	1	0.9	0.497	0.059–4.169	0.429	2.415	0.391		14.921	0.343
Major congenital malformation	14	1.1	1	0.9	1.168	0.152–8.964	1	1.171	0.210		6.542	0.857
Admission to neonatal unit(n,%)	122	9.4	8	7.4	1.298	0.617–2.731	0.491	0.817	0.336		1.984	0.655
Birthweight (g)(mean, SD)	mean	SD	mean	SD	F value		P					
	3273.5	453.32	3286.7	411.67	0.085		0.771					

DVT, deep vein thrombosis; PE, pulmonary embolism; ICP, intrahepatic cholestasis of pregnancy

## Discussion

Reinfection is difficult to confirm because RT-PCR tests can remain positive for weeks following the resolution of clinical symptoms [19]. Therefore, the current gold standard method of identifying reinfection is the detection of a distinct virus by genome sequencing. However, the availability of routine sequencing capabilities at our hospital is limited. Recently, the CDC released guidance to support public health laboratories in the diagnosis of SARS-CoV-2 reinfection [20]. Specifically, the criteria for reinfection include a positive RT-PCR test > 90 days after the initial test (with a Ct of 33) or a positive RT-PCR test > 45 days after the initial test (with a Ct of 33) accompanied by compatible symptoms or known exposure to the virus. In our study, SARS-CoV-2 reinfection was confirmed by a positive nasopharyngeal RT-PCR (with a Ct of 38) or antigen test performed ≥ 90 days after a previous positive test. All participants had symptoms of COVID-19 and had been in close contact with a person with COVID-19 or in a high-risk setting. The fluorescence immunochromatographic SARS-CoV-2 antigen test (Shenzhen Bioeasy Biotechnology Co., Ltd., Shenzhen, China) has a sensitivity of 93.9% (95% CI, 86.5–97.4%), specificity of 100% (95% CI, 92.1–100%), diagnostic accuracy of 96.1%, and kappa coefficient of 0.9 [21].

PCR and antigen tests are both approved by the Food and Drug Administration to diagnose SARS-CoV-2 infection [22]. Although both are highly specific, RT-PCR is typically more sensitive, and antigen tests may be more likely to give false-positive results. However, among persons with a moderate-to-high pretest probability of infection, which includes symptomatic persons who have had close contact with a person with COVID-19, a positive antigen test confirms SARS-CoV-2 infection. Many study participants had positive antigen tests and symptoms within 2–3 weeks of the lifting of strict COVID-19 control measures in Shanghai in late 2022; therefore, the probability of false-positive results was low. Antigen testing may also yield false-negative results, which could be diminished by repeated antigen testing. During the outbreak in Shanghai, more than two antigen tests were performed on each individual, thereby reducing the false-negative rate.

All participants of our study were unvaccinated. In pregnant women, older age, obesity, multiple pregnancy, and comorbidities such as GDM and HDP have been associated with a higher risk of COVID-19 [18]. Our study did not find an association between these risk factors and reinfection, possibly because of the limited number of reinfection cases. The Omicron variant of the SARS-CoV-2 virus can escape immunity acquired from a prior infection, thereby increasing the risk of reinfection [23]. Other reinfection risk factors include female sex, older age, underlying comorbidities, lack of vaccination, and working in healthcare [24–26].

The 7.7% reinfection rate in the present study was consistent with recent studies from various countries that have reported reinfection rates ranging from 5 to 15% [11, 27]. The reported variation may be related to differences in demographic and economic factors, sanitation, public awareness of COVID-19 prevention measures, health policies, and vaccination coverage [11, 27, 28].

All pregnant women in the present study had mild COVID-19 symptoms; reinfections were milder and of shorter duration than single infections. Consistent with this, a previous study has shown that reinfections have a lower risk of symptomatic infection and severe illness than primary infections [29]. One large study suggested that intensive care unit admission was 76% lower after reinfection than after primary infection [24]. A possible cause of milder symptoms in reinfection could be the difference in the variant of infection. Frank P et al. found that disease severities associated with Alpha, Gamma, and Delta variants are comparable, while Omicron infections are significantly less severe [30]. A recent study provides evidence that disease severity is reduced in pregnant women infected by newer variants such as the Omicron strain compared to women infected with wildtype SARS-CoV-2 at the beginning of the pandemic [31]. After the adjustment of COVID-19 epidemic policy, mainland China experienced two consecutive waves of Omicron variants within a 7-month period, 90% of individuals were infected with Omicron BA.5* variants and 28.26% experienced infection with XBB.1* variants. Compared to BA.5* infections, the XBB.1* infections had significantly milder clinical symptoms, lower viral loads, and shorter durations of virus positivity [32].

SARS-CoV-2 enters pulmonary epithelial cells with the spike-like protein on its surface by binding to angiotensin-converting enzyme 2 (ACE2) [33]. The increased ACE2 expression in placental tissue is responsible for placental infection with SARS-CoV-2 and subsequent placental dysfunction, leading to adverse pregnancy outcomes [34]. A previous study correlated placental lesions in pregnant women with COVID-19 with pregnancy outcomes [35]. Ana Medel-Martinez et al. suggested that the frequency of SARS-CoV-2-positive placental tests may differ based on SARS-CoV-2 variants [36]. However, a prospective case-control study found that SARS-CoV-2 infection during the third trimester did not influence placental histological findings [37]. Glynn et al. provided evidence that histologic lesions in the placenta may differ based on the timing of SARS-CoV-2 infection during pregnancy [38]. Since both SARS-CoV-2 variants and the timing of SARS-CoV-2 infection during pregnancy may influence the placenta, whether the times of SARS-CoV-2 infections has an effect on the placenta requires further study.

This study is the first to report the frequency of adverse obstetric outcomes in women with confirmed SARS-CoV-2 reinfection during pregnancy and compare them with those in women with a single SARS-CoV-2 infection. This was a single-center study, the main limitation of which is **its** retrospective design. However, because of the rapid onset of the pandemic and the short duration of the study, this may have decreased recall bias. Additionally, a few women were identified as having SARS-CoV-2 reinfection by repeated positive antigen tests without RT-PCR confirmation. Moreover, the viral variant was not determined in our study; therefore, the influence of viral variants on our conclusion cannot be ruled out. Furthermore, we could not address the influence of vaccination and severe symptoms of COVID-19 on the pregnancy outcome because all our participants were unvaccinated, and all had mild symptoms. Meanwhile, it is possible that our study was underpowered due to the small number of patients. Further large-scale prospective studies are needed to corroborate our findings. Finally, the control group in our study comprised women with first SARS-CoV-2 infection. The lack of healthy pregnant women controls limited our conclusion.

## Conclusion

From the end of 2022, when the strict COVID-19 control measures in China were lifted, we observed a reinfection rate of 7.7% during pregnancy among women at our hospital. Reinfection resulted in milder symptoms compared to single infections, especially in terms of fever. This study found no evidence that SARS-CoV-2 reinfection with mild symptoms increases the risk of adverse obstetric outcomes compared with initial SARS-CoV-2 infections.

## Data Availability

The datasets analysed during the current study are not publicly available due privacy policy but are available from the corresponding author on reasonable request.

## Abbreviations

ACE2: angiotensin-converting enzyme 2
ART: assisted reproductive technology
BMI: body mass index
CDC: Centers for Disease Control and Prevention
COVID-19: coronavirus disease 2019
Ct: cycle threshold
DVT: deep vein thrombosis
FGR: fetal growth restriction
GDM: gestational diabetes
HDP: hypertensive disorders of pregnancy
ICP: intrahepatic cholestasis of pregnancy
PE: pulmonary embolism
RT-PCR: reverse transcription-polymerase chain reaction
SARS-CoV-2: severe acute respiratory syndrome coronavirus 2

## References

[1] Allotey J, Stallings E, Bonet M, Yap M, Chatterjee S, Kew T (2020). Clinical manifestations, risk factors, and maternal and perinatal outcomes of coronavirus disease 2019 in pregnancy: living systematic review and meta-analysis. BMJ.

[2] DeBolt CA, Bianco A, Limaye MA, Silverstein J, Penfield CA, Roman AS et al. Pregnant women with severe or critical corona-virus disease 2019 have increased composite morbidity compared with nonpregnant matched controls. Am J Obstet Gynecol. 2021;224(5):510.e1-510.e12. 10.1016/j.ajog.2020.11.022 PMC7677036 33221292

[3] Hantoushzadeh S, Shamshirsaz AA, Aleyasin A, Seferovic MD, Aski SK, Arian SE (2020). Maternal death due to COVID-19. Am J Obstet Gynecol.

[4] Delahoy MJ, Whitaker M, O’Halloran A, Chai SJ, Kirley PD, Alden N (2020). Characteristics and maternal and birth outcomes of hospitalized pregnant women with Laboratory-confirmed COVID-19 - COVID-NET, 13 States, March 1-August 22, 2020. MMWR Morb Mortal Wkly Rep.

[5] Villar J, Ariff S, Gunier RB, Thiruvengadam R, Rauch S, Kholin A (2021). Maternal and neonatal morbidity and mortality among pregnant women with and without COVID-19 infection: the INTERCOVID multinational cohort study. JAMA Pediatr.

[6] Gurol-Urganci I, Jardine JE, Carroll F, Draycott T, Dunn G, Fremeaux A et al. Maternal and perinatal outcomes of pregnant women with SARS-CoV-2 infection at the time of birth in England: national cohort study. Am J Obstet Gynecol. 2021;225(5):522.e1-522.e11. 10.1016/j.ajog.2021.05.016 PMC8135190 34023315

[7] Lindsay L, Calvert C, Shi T, Carruthers J, Denny C, Donaghy J (2023). Neonatal and maternal outcomes following SARS-CoV-2 infection and COVID-19 vaccination: a population-based matched cohort study. Nat Commun.

[8] Iannaccone A, Gellhaus A, Reisch B, Dzietko M, Schmidt B, Mavarani L (2024). The importance of vaccination, variants and Time Point of SARS-CoV-2 infection in pregnancy for Stillbirth and Preterm Birth Risk: an analysis of the CRONOS Register Study. J Clin Med.

[9] Badr DA, Picone O, Bevilacqua E, Carlin A, Meli F, Sibiude J (2021). Severe Acute Respiratory Syndrome Coronavirus 2 and pregnancy outcomes according to gestational age at Time of Infection. Emerg Infect Dis.

[10] To KK, Hung IF, Ip JD, Chu AW, Chan WM, Tam AR (2021). Coronavirus Disease 2019 (COVID-19) re-infection by a phylogenetically distinct severe Acute Respiratory Syndrome Coronavirus 2 strain confirmed by whole genome sequencing. Clin Infect Dis.

[11] Guedes AR, Oliveira MS, Tavares BM, Luna-Muschi A, Lazari CDS, Montal AC (2023). Reinfection rate in a cohort of healthcare workers over 2 years of the COVID-19 pandemic. Sci Rep.

[12] National Center for Immunization and Respiratory Diseases (U.S.). Division of Viral Diseases. Investigative Criteria for Suspected Cases of SARS-CoV-2 Reinfection (ICR). Oct. 27, 2020. Available online: https://stacks.cdc.gov/view/cdc/96072.

[13] Abu-Raddad LJ, Chemaitelly H, Bertollini R, National Study Group for COVID-19 Epidemiology (2021). Severity of SARS-CoV-2 reinfections as compared with primary infections. N Engl J Med.

[14] Bowe B, Xie Y, Al-Aly Z (2022). Acute and postacute sequelae associated with SARS-CoV-2 reinfection. Nat Med.

[15] Wu Z, McGoogan JM (2020). Characteristics of and important lessons from the Coronavirus Disease 2019 (COVID-19) outbreak in China: Summary of a report of 72 314 cases from the Chinese Center for Disease Control and Prevention. JAMA.

[16] Kiserud T, Piaggio G, Carroli G, Widmer M, Carvalho J, Jensen LN (2021). Correction: the World Health Organization fetal growth charts: a multinational longitudinal study of Ultrasound biometric measurements and estimated fetal weight. PLoS Med.

[17] Brown MA, Magee LA, Kenny LC, Karumanchi SA, McCarthy FP, Saito S (2018). Hypertensive disorders of pregnancy: ISSHP classification, diagnosis, and Management recommendations for International Practice. Hypertension.

[18] Epelboin S, Labrosse J, De Mouzon J, Fauque P, Gervoise-Boyer MJ, Levy R (2021). Obstetrical outcomes and maternal morbidities associated with COVID-19 in pregnant women in France: a national retrospective cohort study. PLoS Med.

[19] Zhou B, She J, Wang Y, Ma X (2020). Duration of viral shedding of discharged patients with severe COVID-19. Clin Infect Dis.

[20] Centers for Disease Control and Prevention. 2020. Common investigation protocol for investigating suspected SARS-CoV-2 reinfection. https://www.cdc.gov/coronavirus/2019-ncov/php/reinfection.html. Accessed 3November 2020.

[21] Porte L, Legarraga P, Vollrath V, Aguilera X, Munita JM, Araos R (2020). Evaluation of a novel antigen-based rapid detection test for the diagnosis of SARS-CoV-2 in respiratory samples. Int J Infect Dis.

[22] Drain PK (2022). Rapid Diagnostic Testing for SARS-CoV-2. N Engl J Med.

[23] Altarawneh HN, Chemaitelly H, Hasan MR, Ayoub HH, Qassim S, AlMukdad S (2022). Protection against the Omicron variant from previous SARS-CoV-2 infection. N Engl J Med.

[24] Mensah AA, Lacy J, Stowe J, Seghezzo G, Sachdeva R, Simmons R (2022). Disease severity during SARS-COV-2 reinfection: a nationwide study. J Infect.

[25] Rivelli A, Fitzpatrick V, Blair C, Copeland K, Richards J (2022). Incidence of COVID-19 reinfection among midwestern healthcare employees. PLoS ONE.

[26] Nordström P, Ballin M, Nordström A (2022). Risk of SARS-CoV-2 reinfection and COVID-19 hospitalisation in individuals with natural and hybrid immunity: a retrospective, total population cohort study in Sweden. Lancet Infect Dis.

[27] Eythorsson E, Runolfsdottir HL, Ingvarsson RF, Sigurdsson MI, Palsson R (2022). Rate of SARS-CoV-2 Reinfection during an Omicron Wave in Iceland. JAMA Netw Open.

[28] Armstrong JN, Campbell L, Rabatsky-Her T, Leung V, Parikh S (2021). Repeat positive SARS-CoV-2 RNA testing in nursing home residents during the initial 9 months of the COVID-19 pandemic: an observational retrospective analysis. Lancet Reg Health Am.

[29] Brouqui P, Colson P, Melenotte C, Houhamdi L, Bedotto M, Devaux C (2021). COVID-19 re-infection. Eur J Clin Invest.

[30] Esper FP, Adhikari TM, Tu ZJ, Cheng YW, El-Haddad K, Farkas DH (2023). Alpha to Omicron: Disease Severity and Clinical outcomes of Major SARS-CoV-2 variants. J Infect Dis.

[31] Serra FE, Rosa Junior ER, de Rossi P, Francisco RPV, Rodrigues AS. COVID-19: impact of original, Gamma, Delta, and Omicron variants of SARS-CoV-2 in Vaccinated and unvaccinated pregnant and Postpartum women. Volume 10. Vaccines; 2022. p. 2172. 10.3390/vaccines10122172 PMC9786095 36560582

[32] Qu L, Xie C, Qiu M, Yi L, Liu Z, Zou L (2024). Characterizing infections in two epidemic waves of SARS-CoV-2 Omicron variants: a Cohort Study in Guangzhou, China. Viruses.

[33] Jing Y, Run-Qian L, Hao-Ran W, Hao-Ran C, Ya-Bin L, Yang G (2020). Potential influence of COVID-19/ACE2 on the female reproductive system. Mol Hum Reprod.

[34] Li M, Chen L, Zhang J, Xiong C, Li X (2020). The SARS-CoV-2 receptor ACE2 expression of maternal-fetal interface and fetal organs by single-cell transcriptome study. PLoS ONE.

[35] Arora D, Rajmohan KS, Singh S, Nair V, Barui S, Dey M (2022). Correlation between placental histopathology and perinatal outcome in COVID-19. Tzu Chi Med J.

[36] Medel-Martinez A, Paules C, Peran M, Calvo P, Ruiz-Martinez S, Ormazabal Cundin M (2023). Placental Infection Associated with SARS-CoV-2 wildtype variant and variants of concern. Viruses.

[37] Tasca C, Rossi RS, Corti S, Anelli GM, Savasi V, Brunetti F (2021). Placental pathology in COVID-19 affected pregnant women: a prospective case-control study. Placenta.

[38] Glynn SM, Yang YJ, Thomas C, Friedlander RL, Cagino KA, Matthews KC (2022). SARS-CoV-2 and placental Pathology: malperfusion patterns are dependent on timing of infection during pregnancy. Am J Surg Pathol.

